# Bioradiotherapy with Cetuximab May Reduce the Risk of Neck Node Relapse in Locoregionally Advanced Laryngeal Glottic Carcinoma: May HER1-Profile Be Useful in the Bioselection of Patients?

**DOI:** 10.3390/jpm12091489

**Published:** 2022-09-11

**Authors:** Giovanni Almadori, Antonella Coli, Eugenio De Corso, Stefano Settimi, Dario Antonio Mele, Francesca Brigato, Domenico Scannone, Jacopo Galli, Vincenzo Valentini, Gaetano Paludetti, Libero Lauriola, Franco Oreste Ranelletti

**Affiliations:** 1 Unit of Head and Neck Surgical Oncology, Unit of Otorhinolaryngology, Fondazione Policlinico Universitario A.Gemelli IRCCS, 00168 Rome, Italy; 2 Department of Head, Neck and Sensory Organs, Unit of Otorhinolaryngology, Università Cattolica del Sacro Cuore, Fondazione Policlinico Universitario A.Gemelli IRCCS, 00168 Rome, Italy; 3 Unit of Anatomic Pathology, Fondazione Policlinico Universitario A.Gemelli IRCCS, 00168 Rome, Italy; 4 Unit of Oncologic Radiotherapy, Fondazione Policlinico Universitario A.Gemelli IRCCS, 00168 Rome, Italy; 5 Unit of Histology, Università Cattolica del Sacro Cuore, 00168 Rome, Italy

**Keywords:** radiotherapy, cetuximab, upfront surgery, HER1 expression, laryngeal cancer

## Abstract

The aim of the study was to evaluate survival in patients with advanced glottic laryngeal squamous cell carcinoma treated by bioradiotherapy (BioRT) with cetuximab and eventual salvage surgery (group A, *n* = 66) or upfront surgery (total laryngectomy or near-total laryngectomy) with or without postoperative radiotherapy (PORT) (group B, *n* = 66). The predictive role of HER1 expression in the bioselection of tumors was evaluated. Relapse-free (RFS), metastasis-free (MFS), overall (OS) survivals, salvageability, and rates of larynx preservation were analyzed. The two groups were balanced by propensity score method on their baseline characteristics. No significant differences in RFS and OS were found, while MFS results were significantly higher in group A (*p* = 0.04). Group A showed a 22% reduction in the probability of nodal metastasis (*p* = 0.0023), mostly in tumors with higher HER1 expression. The salvageability with TL at 3 years was 54% after prior BioRT and 18% after prior upfront NTL (*p* < 0.05). BioRT with cetuximab showed a reduction in the risk of lymph node relapse, particularly in the case of HER1 positive tumors, and it allowed to achieve a higher rate of functional larynx preservation and a higher salvageability compared with upfront surgery. HER1 analysis could be clinically useful in the bioselection of tumors that may benefit from BioRT with cetuximab, particularly in those with neck node metastatic propensity.

## 1. Introduction

Laryngeal squamous cell carcinoma (LSCC) remains one of the most common tumors of the head and neck region, comprising 30% of all cases. Even today, about 60% of patients show a locoregionally advanced disease at diagnosis, becoming one of the few tumors in which the 5-year survival rate has decreased over the past 40 years, even if the overall incidence is declining and the preservation of a functional larynx can be achieved in >50% of patients [1,2,3]. Unlike tumors that arise in the oropharynx, which increasingly are linked to prior infection by human papillomavirus (HPV), tumors of the larynx, oral cavity, and hypopharynx are still primarily associated with tobacco consumption, alcohol abuse, or both and are now collectively referred to as HPV-negative tumors. Treatment of LSCC remains a challenge: while surgery combined with radiotherapy (RT) is the preferred treatment in locoregionally advanced oral cavity cancer, surgery remains a treatment option in cancers of the oropharynx, hypopharynx, and larynx. In fact, in the case of unresectable disease, and because of an emergent ambition to preserve affected organs and their function, definitive RT sometimes represents the treatment of choice. Thus, over time, the decision making changed from survival at all costs to survival with maximum functional outcomes, with a fine balance between overall survival, larynx function preservation, and quality of life. Different nonsurgical larynx preservation strategies, using RT with concurrent cisplatin, induction chemotherapy followed by radiotherapy, or RT alone, were increasingly proposed [1,4,5,6].

Since its approval, the epidermal growth factor receptor (EGFR) antibody cetuximab, in combination with RT, has been increasingly used to treat patients with HNSCC, especially when platinum-based chemotherapy is contraindicated because of toxicity [7], in the elderly, or in addition to cisplatin or anti-PD1 in recurrent disease. Recently, in advanced HNSCC, it was observed that, in comparison with conventional RT, bioradiotherapy with cetuximab significantly improves locoregional control rates and overall survival (OS) without any increase in unmanageable toxicity [8,9,10]. However, no definitive evidence supports the superiority of concurrent cetuximab and RT compared with concurrent platinum-based regimens, and their efficacy remains unclear [9,11,12,13,14,15,16,17,18]. Furthermore, because of retrospective studies from comparable patient groups and settings indicating inferior disease control of RT plus cetuximab compared with RT plus cisplatin [19], patients inclusion in recent trials [15,20] was prematurely closed, and the primary endpoints OS did not reach statistical significance between the treatment groups in the first analysis of the study. The main limit of several previous studies on bioradiotherapy with cetuximab is the assessment of its efficacy on all head and neck squamous cell carcinomas (HNSCCs) regardless of the HPV status [15,20,21,22,23,24,25]. HPV-positive or HPV-negative tumors, in fact, exhibit distinct differences in gene expression and immune profiles, and the inclusion of all HNSCCs underscores the unique biology and heterogeneity of this oncological disease. Effective personalized medicine approaches, including the evaluation of biomarker-driven targeted therapies, are lacking in this disease. Detailed molecular characterization, as well as immune profiling of LSCC, suggests that the incorporation of new prognostic and predictive biomarkers into clinical management may overcome obstacles to targeted therapies and enable prolonged survival [26]. Additional studies are needed to identify more homogeneous subgroups of tumors with the molecular characterization that may benefit from concomitant cetuximab treatment. Because of the ethical restriction in conducting clinical trials on the efficacy of cetuximab in HPV-negative tumors, we conducted a retrospective analysis of two balanced homogeneous series of locoregionally advanced glottic LSCC patients, respectively treated with bioRT with cetuximab and salvage surgery or upfront surgery with postoperative radiotherapy (PORT), taking into account also the clinical relevance and prognostic role of HER1 expression. The primary objective of this study was to investigate OS in patients treated with RT plus cetuximab compared with upfront surgery. Secondary objectives were to compare locoregional control in terms of RFS and MFS, the pattern of failure, and salvageability, and the tertiary objective was to evaluate the prognostic role of tumoral EGFR expression to select a subgroup of LSCC patients that might effectively be targeted with cetuximab. To avoid potential confounding and selection biases, depending on the retrospective design of the study, the two groups of treated patients were balanced by propensity score (PS) method, 35 on their baseline characteristics, including HER1 expression in their tumors. SCCs of the oral cavity, hypopharynx, and larynx are still primarily associated with smoking and alcohol and are now collectively referred to as HPV-negative and generally with a more unfavorable prognosis than HPV-positive HNSCC.

## 2. Materials and Methods

### 2.1. Study Population and Study Design

This is a retrospective observational study of two groups of 66 consecutive untreated primary locoregionally advanced glottic LSCC patients (cT3-T4; unfavorable, local-extended cT2) treated with BioRT with cetuximab with curative intent (Group A) or upfront total laryngectomy (TL) or near-total laryngectomy (NTL) with or without PORT (Group B). Because of the heterogeneity in clinical behavior of larynx subsites, we excluded all patients with primary supraglottic LSCC. Both groups included patients admitted to the Department of Otorhinolaryngology-Head and Neck Oncology between 1999 and 2005 (Institutional Review Tumor Board “SpiderNet”). Patients were eligible if they had pathologically confirmed stage III or IV squamous cell carcinoma, measurable disease, no distant metastases, and no prior therapy. 

Group A included patients treated with bioradiotherapy with cetuximab if they were considered suitable for an organ preservation protocol according to international guidelines, or in case of cT4 disease and any N if they refused TL. Cetuximab treatment consisted of an initial of 400 mg/m^2^ and was delivered as a 120 min intravenous infusion; this initial dose was delivered 1 week before the initiation of intensity-modulated radiotherapy and then 250 mg/m^2^ weekly during the radiotherapy treatment; seven weekly infusions were delivered at a dose of 250 mg/m^2^ for a period of 60 min each. 

We delivered 69.96 Gy at 2.12 Gy per fraction to the planning target volume (PTV) encompassing the gross tumor volume, 59.4 Gy at 1.8 Gy per fraction to the PTV of the high-risk clinical target volume (CTV), and 54 Gy at 1.64 Gy per fraction to the PTV of the low-risk CTV. The gross tumor volumes and CTVs were each expanded 3 to 5 mm to generate their respective PTVs. Cervical lymph node drainage regions, considered to be at high risk for subclinical disease, were treated with a dose of 50 to 54 Gy. The primary tumor and gross nodal disease received full-dose radiotherapy (70–76.8 Gy), depending on fractionation. In the case of histologically proven persistent or recurrent locoregional disease, salvage surgery (total laryngectomy with or without neck node dissection) was performed.

Group B included patients who underwent upfront surgical treatment of the primary tumor related to the lesion extension and location, modified radical neck dissection (MRND) in case of lymph node involvement at clinical presentation (cN+), and observation under strict follow-up conditions with surgical salvage for neck node relapse in clinically negative (cN0) neck tumors. We performed a TL in all 35 out of 66 cT3 and cT4 tumors. Regarding the subgroup of 31 out of 66 patients with unfavorable cT2 limited-extended tumors, in 15 out of 31 patients, we performed an NTL (cricohyoidopexy) because of large tumor volume, deep-tissue invasion, and the involvement of the anterior commissure, whereas in the remaining 16, including 8 cN+ tumors, we performed a TL because the vocal cord impaired mobility for deeper tissue invasion, subglottic extension, involvement of cricoarytenoid unit or posterior commissure, and poor compliance and tolerance to NTL. In summary, 51 out of 66 patients underwent TL, and 15 out of 66 underwent NTL. 

Regarding adjuvant treatment in Group B, PORT (60–70 Gy, 180 cGy per fraction) on primary and nodal echelons with or without chemotherapy (q21 cisplatin) was administered in cases of locally advanced tumors (pT4), positive or close resection margins, pN1 extranodal spread, and pN2–N3 disease. In all cases of locoregional recurrence, secondary salvage surgery (TL or neck surgery) was performed. 

All patients were initially evaluated with a comprehensive head and neck examination, which included fiberoptic endoscopy. Initial evaluation included computed tomography or magnetic resonance imaging of the head and neck region and chest radiography or CT scan. Histopathological grading was independently assessed by two pathologists, according to WHO guidelines [27]. After the workup, all cases were staged and discussed by the tumor board involving at least a medical oncologist, a radiation therapist, and a head and neck surgeon. Post-treatment assessments were performed 4 and 8 weeks after completion of radiotherapy or surgery. Subsequently, patients were evaluated every 3 months during the first and second years and every 6 months during years 3 to 5. This follow-up assessment included physical examination with panendoscopy and imaging studies consisting of computed tomography or magnetic resonance imaging of the head and neck region and chest CT scan. Baseline characteristics are reported in Table 1. 

### 2.2. Immunohistochemical Analysis

HER1 expression in tumor cells was evaluated by immunohistochemistry (IHC) on formalin-fixed and paraffin-embedded tumor tissues, according to standard procedures. Tumor samples from both groups of patients were processed by the same standardized IHC procedures, utilizing anti-HER1 (clone H11, dilution 1:150; Dako, Milano, Italy) monoclonal antibody; the procedure for quantizing the results was previously reported [26].

### 2.3. Statistical Analysis

**Figure 1:**
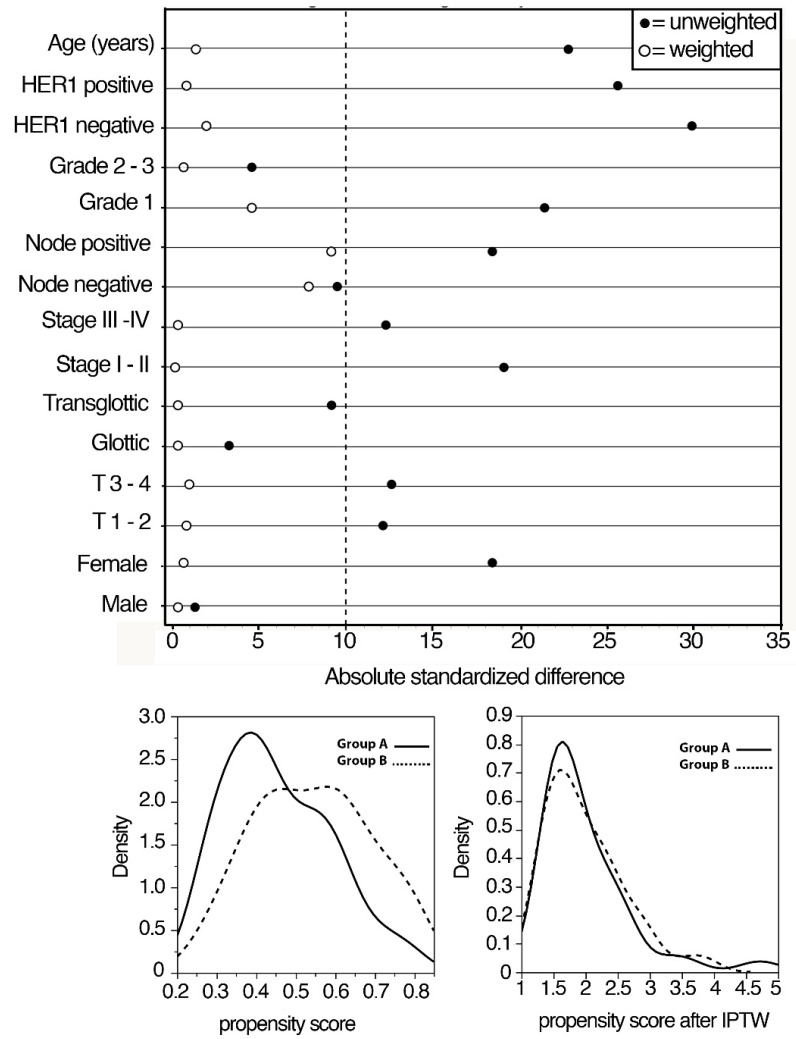
Absolute standardized differences in unweighted and inverse propensity score weighted samples.

We used the PS method to avoid potential confounding and selection biases depending on the retrospective design of the study. The selection of the covariates for balancing the two groups was made according to the results of previous studies from our cohort investigating prognostic factors in locally advanced LSCC patients. Logistic regression was used to estimate PS, representing the probability of being treated with upfront surgery or cetuximab + RT, given the individual’s covariates. A detailed procedure of the PS method is reported in Figure 1.

All medians and life tables were computed by adjusted Kaplan–Meier estimates of survival curves in the sample weighted by the inverse probability of treatment weighted (IPTW). The curves were compared by a weighted version of the log-rank test [28]. Adjusted hazards were calculated by IPTW Cox proportional hazards regression, using a robust variance estimator for inference [29]. The proportional hazards assumption and collinearity were verified by Schoenfeld residuals and variance inflation factors, respectively. Sensitivity analysis was, therefore, performed.

Regarding the primary endpoint, survival rates were calculated from the date of the first surgery or the beginning of the cetuximab-based concurrent radiotherapy to the date of clinical or pathological local recurrence (RFS), or neck node recurrence (MFS), death regardless of the cause (OS), or to the date of the last available information on the patient’s status. For the secondary endpoint, OS was calculated from the date of salvage surgery to the date of death regardless of the cause or the date of the last available information on the patient’s status. Two-sided *p* < 0.05 was considered significant in statistical tests. Analyses were performed using the JMP version 13.2 (SAS Institute Inc., Cary, NC, USA) and R software version 3.3.341.

## 3. Results

### 3.1. Survival Analysis According to Primary Treatments 

Unweighted Kaplan–Meier analysis revealed that MFS was significantly higher in Group A (94%) compared with Group B (74%) (*p* = 0.04), while no significant differences were found in the RFS (*p* = 0.32) and OS (*p* = 0.25) (Table 2). 

**Figure 2:**
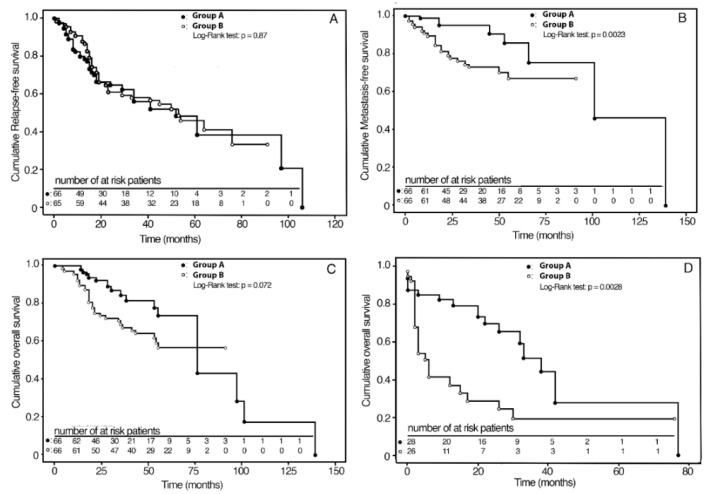
Kaplan–Meier analyses of survival curves for therapy weighted by inverse probability for baseline confounding variables; (**A**) 3-year RFS in groups A and B; (**B**) 3-year MFS in groups A and B; (**C**) 5-year OS in groups A and B; (**D**) 3-year OS in groups A and B after salvage surgery.

Kaplan–Meier survival curves in the IPTW adjusted samples are shown in Figure 2A–C. The RFS and OS curves did not differ between the two groups. At the 3-year follow-up, the cumulative MFS rates were 95% (C.I. 95%: 87–99) and 73% (C.I. 95%: 60–83) for Group A and Group B, respectively. 

Group A, compared with Group B, showed a 22% (C.I. 95%: 9–35) absolute reduction in the probability of nodal metastasis (*p* = 0.0023) (Table 2). Survival rates according to the stage are reported in Table 3. In particular, for stage II, MFS was 100% and 86% (C.I. 95%: 72–100) in Group A and in Group B (*p* = 0.07), respectively, with an absolute risk reduction in nodal metastasis of 14% (C.I. 95%: 1–28). For Stage III-IV, MFS was 92% (C.I. 95%: 83–100) in Group A and 63% (C.I. 95%: 45–79) in Group B (*p* = 0.002), with an absolute risk reduction in nodal metastasis of 29% (C.I. 95%: 12–48), two times greater than stage II. For Group A patients, sensitivity analysis with IPTW weighted Kaplan–Meier without outliers showed a similar reduction (20%: C.I. 95%: 6–34) in the probability of metastasis (*p* = 0.0038). As regards OS and RFS, significant differences were not observed. 

### 3.2. Survival Rates According to HER1 Expression

The effect of BioRT with cetuximab on survival rates was evaluated according to tumor HER1 expression. The 3y-MFS for HER1-positive patients was 89% (C.I. 95%: 76–98) in Group A and 59% (C.I. 95%: 42–78) in Group B (*p* = 0.01), with an absolute risk reduction in nodal metastasis of 30% (C.I. 95%: 6–50). In the HER1 negative patients, 3y-MFS was 99% (C.I. 95%: 96–100) in Group A and 87% (C.I. 95%: 76–98) in Group B (*p* = 0.04). Compared with Group B, Group A patients showed a 12% (C.I. 95%: 2–90) absolute risk reduction in nodal metastasis (Table 4).

### 3.3. Multivariate Analysis

IPTW weighted Cox regression revealed that transglottic tumor site and HER1 positivity behaved as independent prognostic factors of reduced RFS (concordance index = 0.72). HER1 tumor positivity and up-front surgery with or without PORT were independent prognostic factors of reduced MFS (concordance index = 0.81). Finally, HER1 tumor positivity, up-front surgery with or without PORT, age, and positive nodal status behaved as independent prognostic indicators of reduced OS (concordance index = 0.79) (Table 5). 

Sensitivity analysis by asymmetrical trimming supports the robustness of the statistical inference. Moreover, to invalidate the inference, 71/132 (54%) of the observations would have to be replaced with cases for which the effect should be 0.

### 3.4. Analyses of Oncological Results after Salvage Surgery

All patients of Group A who relapsed (28 out of 66) underwent salvage TL, with or without unilateral or bilateral therapeutic neck dissection. In Group B, 27 out of 66 patients locally relapsed and were treated as follows: 10 out of 15 patients underwent salvage TL after previous NTL; 17 out of 51 patients underwent salvage neck surgery after previous TL; all these patients underwent unilateral or bilateral therapeutic neck dissection. In our analysis, we also observed a high rate of functional laryngeal preservation (LP) with BioRT with cetuximab. More precisely, in Group A, 38 out of 66 patients (57.6%) saved their functional larynx in place, while in Group B, only 5 out of 15 patients who underwent upfront NTL (33.3%) preserved it. Furthermore, overall salvageability was better after BioRT with cetuximab compared with upfront surgery failures; at 3-year follow-up after salvage surgery, OS was 54% (C.I. 95%: 29–79) and 18% (C.I. 95%: 3–34) for Group A and Group B, respectively. Compared with Group B, Group A patients showed a 36% (C.I. 95%: 7–65) absolute reduction in death risk (*p* = 0.014) after salvage surgery. Kaplan–Meier survival curves in the IPTW adjusted samples are shown in Figure 2D. The mean times elapsed from relapse to death were 24.2 and 7.1 months for patients of Group A and Group B, respectively (*p* = 0.0028). 

## 4. Discussion

To the best of our knowledge, this is the largest retrospective study assessing the survival in two homogeneous groups of patients with locoregionally advanced glottic LSCC treated by BioRT with cetuximab compared to patients treated by upfront surgery. Considering that LSCCs from different subsites have distinct presentations and prognoses, in this study, we analyzed only cancers starting from the glottis subsite. Herein, we also used the propensity score (PS) method for weighting patients so that the resulting groups will have similar characteristics to those created through random assignment, balancing the distribution by IPTW estimators on the set of clinical covariates and HER1-expression. 

Precision medicine approaches for patients with HNSCC are lacking. Unlike many other cancer types, HNSCCs are a heterogeneous group of tumors located in the oral cavity, oropharynx, hypopharynx, and larynx characterized by variability in prognosis and molecular profiles. HPV infection probably covers most of the HNSCC heterogeneity. HPV-positive and HPV-negative tumors are distinct subtypes concerning different anatomical locations (oropharynx versus nonoropharynx), molecular signature, clinical presentation, and response to therapy. Similarly, squamous cell carcinomas from different parts of the larynx have distinct presentations and prognoses, but the molecular basis for this discrepancy has yet to be characterized. Recently, significant genomic, transcriptomic, and proteomic differences between supraglottic and glottic HPV-negative LSCC were demonstrated [30], suggesting that molecular-level differences play important roles in the discrepancies in outcomes between these two subsites. For example, PIK3CA and S6, critical members of the P13K/Akt pathway that in HNSCC is responsible for tumorigenesis, invasion, metastasis, and resistance to anticancer therapy, were found to be more highly expressed in the supraglottic versus the glottis subsite [31,32]. Moreover, PD-L1 proteins were significantly higher in cancers at the glottis subsite, suggesting that glottic cancers may be the more favorable target for immunotherapy alone or in combination with cetuximab than supraglottic cancers [33].

Our study does not have human papillomavirus (HPV) status because it was initially thought that HPV did not play a role in laryngeal cancer [34]. It is estimated that in LSCC, the prevalence of HPV ranges from 20% to 25% [35,36], and its role remains controversial [37]. Correspondingly, EGFR is overexpressed in 80–90% of LSCC tumors, for the first time quantitatively evaluated in fmole/mg/protein on fresh tumor tissue sample in our previous preliminary study [38] and it resulted associated with poor relapse-free survival (RFS) and OS [34], lower metastasis-free survival [39] in specific laryngeal site cancers. Subsequently, lower progression-free survival (PFS) was documented in HNSCC overexpressing EGFR [40,41]. Overexpression of other receptor tyrosine kinases, including HER2, HER3, MPM7, and coexpression of all four HER family member (HER1, HER2, HER3, HER4) receptors, also may contribute to tumor resistance to EGFR-targeting agents [26,42,43,44]. 

It is known that lymph node involvement remains an important independent prognostic factor for all outcomes, including OS, DSS, and RFS. Occult metastases in the draining cervical lymph nodes might be present in patients with locoregionally advanced LSCC, and the use of elective selective neck irradiation or neck dissection improves survival. 

In our study, the comparison of the two therapeutic interventions, both in unweighted and IPWT-weighed samples, revealed that the treatment with BioRT with cetuximab and salvage surgery or upfront surgery with or without PORT had similar effects on OS. Patients undergoing BioRT with cetuximab showed a 22% reduction in the absolute risk of neck node relapse compared with those treated with upfront surgery; this effect was stronger in stages III–IV than in stage II tumors. In our previous studies, a close correlation was observed between the presence of high HER1 expression and a lower MFS; moreover, analysis of logarithmically transformed HER1 values showed that the risk of neck node relapse increased significantly with higher HER1 values, resulting in HER1 expression as an independent predictor of higher metastatic propensity [34,39]. The latter, in fact, is a prognostic factor of the clinical outcome [42], a biomarker of resistance to RT [45], and a target of cetuximab action [26,46]. 

The results from a randomized controlled trial [47] have suggested a positive predictive value of high HER1 expression in locoregional tumor control by continuous hypo-fractionated accelerated radiotherapy. Accordingly, we observed that the effect on the absolute reduction in the probability of tumor cell invasion and neck node metastasis by BioRT with cetuximab was greater in patients with higher HER1 expression. 

The effect of the combined therapy on reducing the intrinsic biological invasiveness of tumor cells and neck node metastatic propensity could be explained by the efficacy of cetuximab in reducing radioresistance of tumor cells by inhibiting the action of HER1 [48] and the radiation-induced upregulation of HIF-1α [49]. Moreover, this effect is consistent with the observation that the addition of cetuximab to high-dose radiotherapy significantly increases the control of regional and distant metastatic disease and survival in patients with locally advanced HNSCC, especially in nonoropharyngeal HPV-negative and HER1-positive tumors, without increasing radiation-associated acute toxicity [8,50]. HER1 can induce epithelial–mesenchymal transition (EMT) in HNSCC cell lines through PI3K/Akt signaling [31,32]. Based on this evidence, it can be hypothesized that cetuximab can contribute to reducing the cellular spread and invasion by inhibiting the HER1 promoting the EMT and endowing the stem-like phenotype of primitive tumor cells. Moreover, an independent correlation of HER family members with the presence of nodal metastases and poor clinical outcomes has been reported [51]. A significant role of the cooperative signaling of all four HER receptor members in the metastatic potential of LSCC [42] is the most widely accepted hypothesis.

In our analysis, we also observed a higher rate of laryngeal preservation in patients treated with BioRT with cetuximab compared with the patients who underwent NTL; furthermore, salvageability was also superior in the patients with failure after prior BioRT than in those with failure after prior upfront NTL. Our observation of a greater benefit of cetuximab treatment in Stages III–IV than in Stage II cancers rules out the possibility that the overall efficacy of cetuximab can be sustained by the inclusion of Stage II tumors. Even though the optimal larynx preservation strategy based on tumor bioselection still remains to be defined, the potential role of cetuximab or other molecular targeting agents, alone or in combination with small molecules or immunotherapy, is encouraging. Besides the inherent limitations of this study, rooted in the retrospective nature of our analysis and limited sample size, we used real-world data (Spider platform) from a multidisciplinary clinical practice in a single comprehensive cancer center with the same philosophy.Besides the inherent limitations of this study, rooted in the retrospective nature of our analysis and limited sample size, we used real-world data (Spider platform) from a multidisciplinary clinical practice in a single comprehensive cancer center with the same philosophy.

## 5. Conclusions

Further evaluation in a larger population affected by HPV-negative/HER1-positive/PD-L1-positive tumors is necessary to fully assess the potential value of cetuximab alone or in combination with immunotherapy to inhibit the process of tumor cell invasion and regional and distant metastasis. The development of novel clinical trial platforms, in conjunction with new targeted agents and with immune profiling approaches to translate molecular findings to clinical benefit, is critical for the successful implementation of precision and personalized larynx cancer medicine. 

## Figures and Tables

**Table 1:** Baseline characteristics of the studied patients.

CHARACTERISTIC	GROUP A (*n* = 66)	GROUP B (*n* = 66)	*p*
FOLLOW-UP (years) Median (range)	30 (5–139)	47 (2–91)	
AGE Median (Range)	65 (40–86)	62 (39–80)	
SEX: Female	5 (8%)	4 (6%)	
Male	61 (92%)	62 (94%)	1.0 ^(1)^
GRGRADE: 1	13 (20%)	10 (15%)	
2	22 (33%)	18 (27%)	
3	31 (47%)	38 (58%)	0.47 ^(2)^
TUMOR SITE: Glottic	47 (71%)	49 (74%)	
Transglottic	19 (29%)	17 (26%)	0.84
T CLASSIFICATION: 2	36 (54%)	31 (47%)	
3	19 (29%)	20 (30%)	
4	11 (17%)	15 (23%)	0.60
STAGE: II	29 (44%)	23 (35%)	
III	15 (23%)	25 (38%)	
IV	22 (33%)	18 (27%)	0.17
NODAL STATUS: 0	45 (68%)	41 (62%)	
1	5 (8%)	15 (23%)	
2	16 (24%)	10 (15%)	0.03
HER1 STATUS: Negative	25 (38%)	36 (55%)	
Positive	41 (62%)	30 (45%)	0.08

^(1)^ Fisher’s exact test; ^(2)^ likelihood ratio test.

**Table 2:** Kaplan–Meier estimates of cumulative survival in patients treated with cetuximab + radiotherapy or with up-front surgery + radiochemotherapy.

KAPLAN–MEIER SURVIVAL ESTIMATES	UNWEIGHTED	*p* ^1^	IPWT-ATE ^2^	ABSOLUTE RISK REDUCTION	*p* ^3^
3-year RFS: Group A	56% (41–70) ^4^		54% (38–70)		
Group B	63% (51–75)	0.32	58% (45–71)	4% (−2–24)	0.72
3-year MFS: Group A	94% (89–100)		95% (87–99)		
Group B	74% (63–85)	0.04	73% (60–83)	22% (9–35)	0.0008
5-year OS: Group A	69% (52–85)		73% (58–87)		
Group B	60% (47–74)	0.25	56% (42–70)	17% (4–36)	0.10

^1^ Log-rank test; ^2^ average treatment effects from Kaplan–Meier estimates adjusted for baseline covariates by IPWT; ^3^ adjusted log-rank test; ^4^ cumulative survival rates (C.I.-95%). RFS—relapse-free survival; MFS—metastasis-free survival; OS—overall survival.

**Table 3:** Kaplan–Meier estimates of the survival rates according to the Stage of LALSCC in patients treated with cetuximab + radiotherapy or with up-front surgery + radiochemotherapy.

	3-Year RFS	3-Year MFS	5-Year OS
Group A	Stage II: 62% (45–79) ^1^	Stage II: 100%	Stage II: 66% (36–96)
	Stage III–IV: 49% (26–72)	Stage III–IV: 92% (83–100)	Stage III–IV: 76% (58–93)
Group B	Stage II: 59% (37–78)	Stage II: 86% (72–100)	Stage II: 61% (40–82)
	Stage III–IV: 57% (40–75)	Stage III–IV: 63% (45–79)	Stage III–IV: 56% (39–72)
Absolute probability reduction	Stage II: 3% (−37–24)	Stage II: 14% (1–28)	Stage II: 5% (−42–31)
	Stage III–IV: 8% (−19–36)	Stage III–IV: 29% (12–48)	Stage III–IV: 20% (5–46)
Adjusted log-rank test *p*=	Stage II: 0.82	Stage II: 0.07	Stage II: 0.78
	Stage III–IV: 0.55	Stage III–IV: 0.002	Stage III–IV: 0.12

^1^ Average treatment effect (C.I. 95%) from Kaplan–Meier estimates adjusted for baseline covariates by IPWT. RFS—relapse-free survival; MFS—metastasis-free survival; OS—overall survival.

**Table 4:** Kaplan–Meier estimates of the marginal effect of cetuximab + RT and up-front surgery therapies on metastasis-free survival rates in two subgroups of patients subdivided according to HER1 status of LSCC.

Kaplan–Meier Survival Estimates	HER1 Positive (*n* = 71)	Absolute Probability Reduction (*p*)	HER1 Negative (*n* = 61)	Absolute Probability Reduction (*p*)
3-year MFS	Group A: 89% (76–98) ^1^ Group B: 59% (42–78)	30% (6–50) *p* ^2^ = 0.01	Group A: 99% (96–100) Group B: 87% (76–98)	12% (2–90) *p* = 0.04

^1^ Average treatment effect from Kaplan–Meier survival estimates adjusted for baseline covariates by IPWT; ^2^ adjusted log-rank test; MFS—metastasis-free survival.

**Table 5:** Multivariable modeling of predictors for relapse-free, metastasis-free, and overall survival in the cohorts of LALSCC patients weighted for the inverse estimates of the probability of Cetuximab + radiotherapy (CTX + RT) or up-front surgery + radiochemotherapy (SURG + RCT) treatments.

	*n*	RFS (RR ^1^-C.I. 95%-*p* ^2^)	MFS (RR- C.I. 95%-*p*)	OS (RR- C.I. 95%-*p*)
Age (per year)	132	0.98 (0.95.−1.00); *p* = 0.11	0.97 (0.26–1.18); *p* = 0.14	0.95 (0.92–0.99); *p* = 0.016
Gender: Female	9	1	1	1
Male	123	2.19 (0.51–9.45); *p* = 0.29	0.55 (0.26–1.18); *p* = 0.12	2.25 (0.49–10.4); *p* = 0.30
Grade: 1	23	1	1	1
2–3	109	1.31 (0.60–2.90); *p* = 0.49	0.70 (0.18–2.61); *p* = 0.59	1.17 (0.34–4.0); *p* = 0.80
Tumor site: glottic	96	1	1	1
transglottic	36	2.39 (1.31–4.34); *p* = 0.043	0.39 (0.11–1.39); *p* = 0.15	2.10 (0.96–4.43); *p* = 0.064
T-classification: 2	67	1	1	1
3–4	65	1.05 (0.48–2.33); *p* = 0.90	3.97 (0.67–23.5); *p* = 0.13	0.62 (0.27–1.45); *p* = 0.27
Stage: II	52	1	1	1
III–IV	80	0.85 (0.37–1.99); *p* = 0.71	1.41 (0.22–8.92); *p* = 0.71	2.10 (0.74–5.80); *p* = 0.17
Nodal status: negative	87	1	1	1
positive	45	1.38 (0.70–2.70); *p* = 0.35	1.81 (0.55–5.90); *p* = 0.33	2.92 (1.24–6.85); *p* = 0.014
HER1 Status: negative	61	1	1	1
positive	71	3.99 (1.97–8.09); *p* = 0.0001	6.71 (2.39–18.8); *p* = 0.0003	5.90(2.41–14.5); *p* = 0.0001
Therapy: CTX + RT	66	1	1	1
Surgery+ PORT	66	0.79 (0.46–1.34); *p* = 0.38	4.98 (1.46–16.9); *p* = 0.010	2.61 (1.32–5.14); *p* = 0.006

^1^ Reference risk; ^2^ Wald test.

## Data Availability

Derived data supporting the findings of this study are available from the corresponding author on request.
